# An atypical presentation of mesenteric bucket-handle injury following direct trauma during football: a case report

**DOI:** 10.1093/jscr/rjag407

**Published:** 2026-05-29

**Authors:** Rakan Mal, Abdulkareem Alshamrani, Ahmed Saggaf, Shadi Sulaimani, Renad Alzahrani

**Affiliations:** Department of General Surgery, King Abdulaziz Hospital, Jeddah, Saudi Arabia; Department of General Surgery, King Abdulaziz Hospital, Jeddah, Saudi Arabia; Department of General Surgery, King Abdulaziz Hospital, Jeddah, Saudi Arabia; Department of General Surgery, King Abdulaziz Hospital, Jeddah, Saudi Arabia; Faculty of Medicine, Ibn Sina University, Jeddah, Saudi Arabia

**Keywords:** mesenteric bucket-handle injury, internal herniation, blunt abdominal trauma, small bowel ischemia, delayed diagnosis

## Abstract

Mesenteric bucket-handle injuries are critical abdominal traumas typically associated with high-impact deceleration mechanisms. This case report describes an atypical presentation involving a 32-year-old healthy male who sustained a direct knee impact during a football match. Initially discharged due to a perceived low-risk mechanism, the patient returned three days later with signs of small bowel obstruction. Diagnostic imaging suggested internal herniation, and surgical exploration revealed a segment of ischemic herniated bowel within a large mesenteric bucket-handle defect. The patient underwent bowel resection with anastomosis and primary repair of the mesenteric defect, resulting in an uneventful recovery. This case underscores the diagnostic challenges of low-energy mechanisms and atypical radiological findings. It emphasizes the need for a high index of clinical suspicion in blunt abdominal trauma to prevent delayed diagnosis and serious ischemic complications.

## Introduction

Mesenteric bucket-handle injury accounts for the majority of missed bowel and mesenteric injuries [1]. It is a traumatic abdominal injury in which ischemia, hollow-viscus perforation, and revascularization can result from avulsion of the mesentery from a segment of the bowel loop [2]. While it has been associated with significant morbidity and mortality, its initial presentation can be vague with delayed onset of symptoms; nausea, vomiting, abdominal pain, also bleeding can be slow, and perforation may occur secondary to ischemia after 2 to 3 days after initial injury impact [3, 4].

Mesenteric bucket-handle injuries results from shearing forces sustained during deceleration injuries, particularly with the use of seat belt [4]. In addition, compression forces arising from bicycle handlebar injuries or from direct force to the abdomen can result in mesenteric bucket-handle injury [4]. The areas vulnerable to bucket-handle tears are the areas between fixed and mobile segments of the bowel, such as the ligament of Treits, with the majority occurring in the proximal jejunum and the distal ileum near the ileocecal valve [5].

While computed tomography (CT) is the recommended imaging modality for patients with blunt abdominal trauma, there are no pathognomonic features for mesenteric bucket-handle injuries [6]. As reported in the literature, up to 58% of mesenteric avulsion injuries could be missed during the initial clinical assessment and imaging [5]. Adding this to the vague initial clinical presentation, it makes mesenteric bucket-handle injuries a challenging diagnosis to make. Let alone if the mechanism of injury was not typical for this kind of injuries. In this case report we present a young athlete who was found to have mesenteric bucket-handle tears causing internal herniation following a knee kick while playing football. This report has been prepared in accordance with the updated consensus Surgical CAse REport guidelines [7].

## Case presentation

We present a 32-year-old previously healthy male who sustained blunt abdominal trauma after a direct knee impact while playing football. The patient presented on the day of injury to the Emergency Department (ER) with abdominal pain, however, due to the mechanism of injury no imaging studies were performed and patient was discharged home with red flags on when to come to ER. The patient presented 3 days later with inability to tolerate oral diet, nausea, vomiting, and absence of bowel motion for 3 days.

On examination, the patient looked well, mildly dehydrated, hemodynamically stable. Abdominal examination revealed no visible bruises on the abdomen or flanks, his abdomen was mildly distended with mild generalized voluntary guarding. Focused assessment with sonography for trauma (FAST) was done and it showed free fluid in the abdomen. Laboratory results revealed no leukocytosis, haemoglobin of 15 g/dl, unremarkable VBG an lactate level and high creatinine of 175 μmol/L.

**Figure 1:**
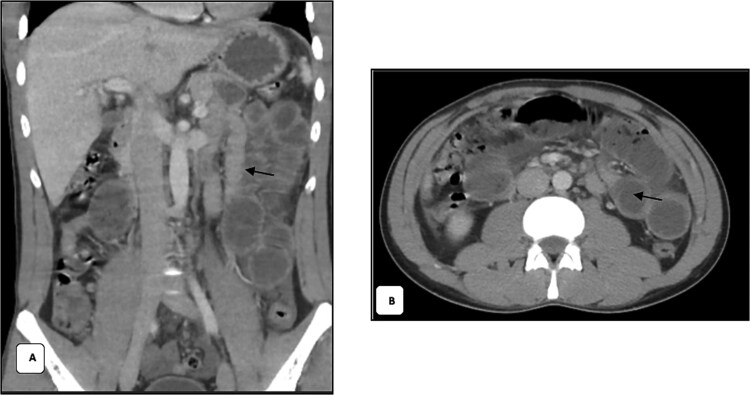
(A) Coronal contrast-enhanced CT shows dilated proximal jejunal loops with air-fluid levels and a clear transition zone in the left lower quadrant (arrow). (B) Axial contrast-enhanced CT shows dilated small-bowel loops with preserved wall enhancement, patent mesenteric vessels and a transition zone in the left lower quadrant (arrow).

Since the patient was stable and had high creatinine level, CT abdomen with IV contrast was only done after following a renal protective protocol. CT abdomen with IV contrast demonstrated dilated jejunal loops with a transitional zone seen at the left lower quadrant, mild abdominal, and pelvic mesenteric fatty stranding and mild free fluid in the abdomen (Fig. 1A and B). The constellations of these findings, raised the suspicion for mesenteric contusion and tear with closed-loop obstruction.

**Figure 2:**
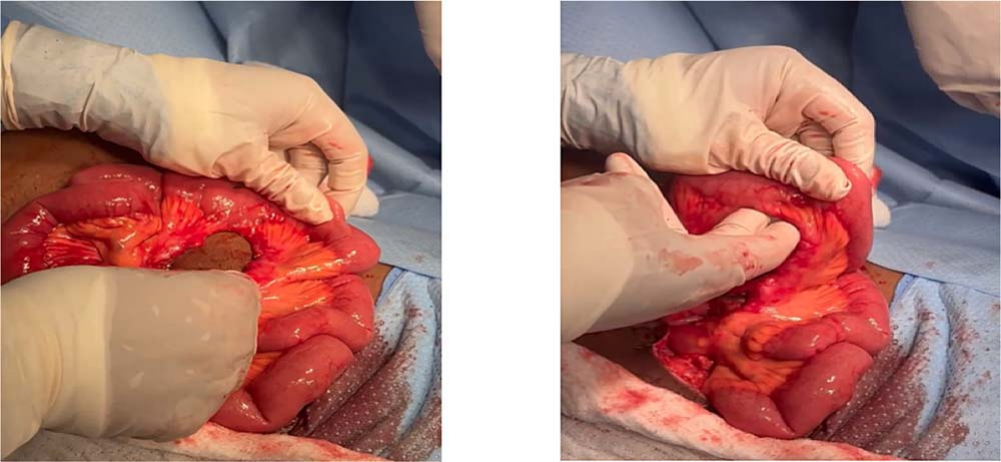
(A) Handle bucket injury with mesentery root defect. (B) The defect measured almost two fingers with 4 × 5 cm (~1.97 in) diameters.

**Figure 3:**
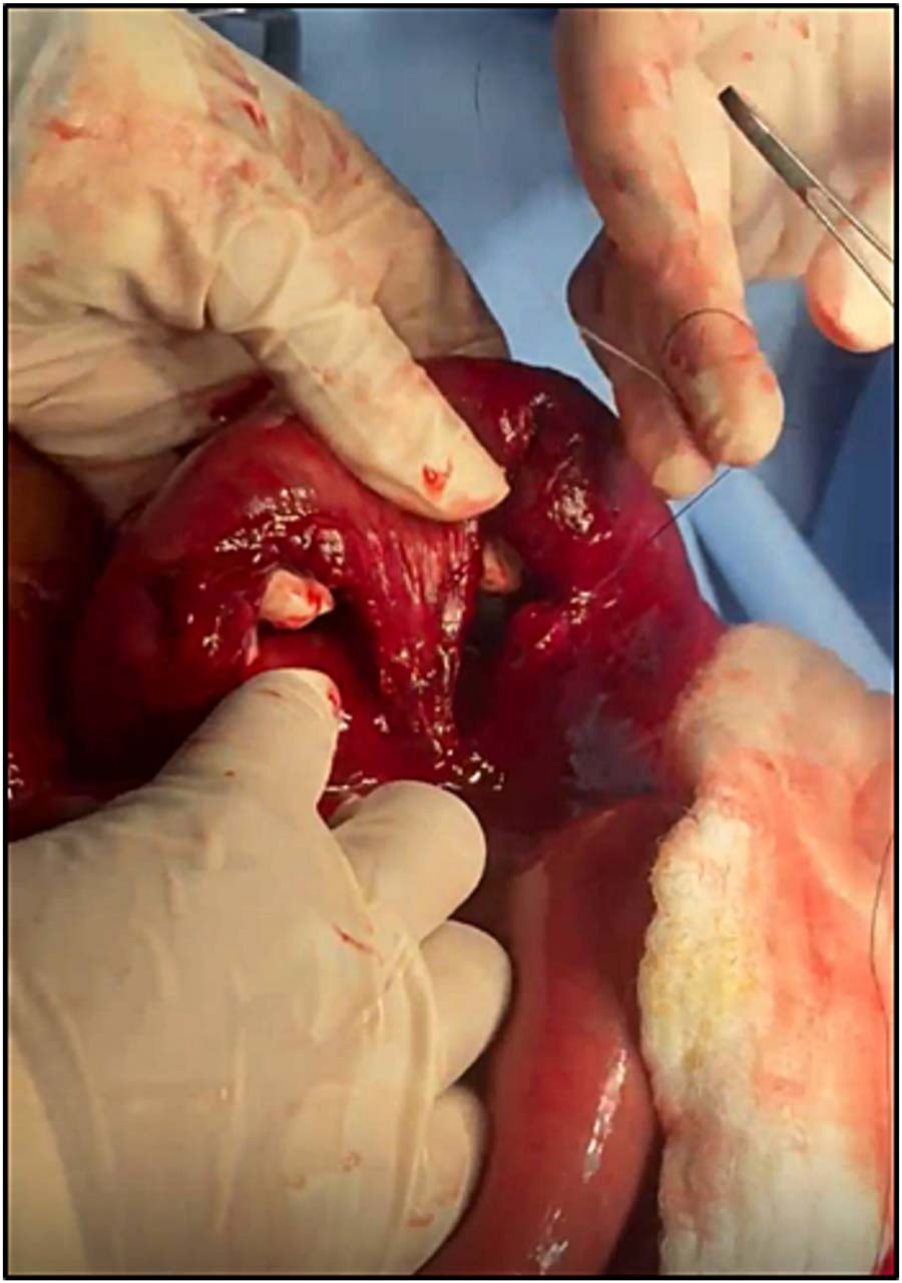
The ischemic segment of the bowel which was found internally herniated and containing two completely avulsed mesenteric defects.

The patient was taken to the operating theatre and procedure started with diagnostic laparoscopy. Laparoscopic exploration revealed sigmoid mesenteric hematoma, multiple mesenteric defects along the mesentery of the small bowel. The procedure was then converted to open laparotomy to address and fully explore these injuries. After running the bowel, a segment of the small bowel ~150–200 cm away from the DJ junction was found internally herniated into another large mesenteric bucket-handle defect located ~250 cm from the DJ junction (Fig. 2). This bowel segment was reduced and it was found dusky even after optimal oxygenation and warming, this segment also contained two completely avulsed mesenteric defects (Fig. 3).

Subsequently, the dusky ischemic segment was resected and a side-to-side anastomosis was performed using gastrointestinal anastomosis stapler. The large mesenteric bucket-handle defect which was the site of internal hernia (Fig. 3) was primarily repaired a prolene suture as no resection was needed since the bowel segment was well vascularized.

Lastly, the sigmoid was inspected, no injuries were found except for mesenteric hematoma which was not explored. A drain was left in the pelvis prior to closure of the abdomen.

The patient had an uneventful postoperative recovery, gradually resumed oral intake, and was discharged on postoperative day five with drain care and follow-up arranged. On 1-week follow up appointment the patient was doing well and the drain was removed.

## Discussion

Mesenteric bucket handle injuries are typically associated with high impact deceleration mechanisms, such as motor vehicle accidents with seatbelt compression, handlebar injuries or falls from height [8]. However, in our case, the mechanism which was a direct knee impact during a football match was atypical and misleading. The absence of a typical deceleration pattern or external bruising likely contributed to the underestimation of the injury at the initial presentation. Consequently, no imaging studies were requested during his initial ER visit, and the patient was discharged with only red flags. This aligns with the literature describing that patients with blunt abdominal trauma who are hemodynamically stable and have vague clinical findings are at high risk of missed bowel or mesenteric injuries [3, 4].

The mechanism of injury in our case represents a localized compression and shear injury rather than the classical high velocity deceleration. This direct trauma created sufficient tensile forces to cause mesenteric tears. This reinforces that even low energy mechanisms should not exclude the possibility of mesenteric bucket handle injuries.

With regards to radiological findings, the CT findings of bucket handle injury in our case were atypical. Rather than showing overt signs such as bowel wall hypoenhancement, mesenteric vascular beading, or extravasation, the scan demonstrated dilated jejunal loops with a transition point in the left lower quadrant, mild mesenteric stranding, and minimal free fluid. These features were more consistent with internal herniation or closed-loop obstruction. The literature confirms that CT remains the modality of choice for blunt abdominal trauma, but with only 45%–75% sensitivity for mesenteric avulsion or devascularizing injuries. Extein *et al*. highlighted that free fluid and mesenteric hematoma are the most frequent findings, while hypoenhancement or discontinuity of mesenteric vessels are less common but more specific [6].

The final intraoperative finding of a devascularized small bowel loop that had herniated through a mesenteric defect validated the initial suspicion of closed-loop obstruction but revealed the underlying mechanism to be a mesenteric avulsion tear. Such secondary internal herniation through traumatic mesenteric defects has rarely been reported, making this presentation distinctly unusual.

This case underscores the importance of maintaining a high index of suspicion for mesenteric injury even in atypical mechanisms and emphasizes that radiological ambiguity should not delay surgical exploration when clinical deterioration ensues.

## Conclusion

This case highlights the importance of maintaining a high index of suspicion for mesenteric bucket handle injuries, even in patients presenting after seemingly minor or atypical blunt abdominal trauma. The misleading mechanism, a localized knee impact without high velocity deceleration led to an underestimation of injury severity and initial mismanagement in the Emergency Department. Furthermore, the atypical CT findings mimicking internal herniation and closed-loop obstruction underscore the diagnostic challenges of these injuries. Timely surgical exploration remains the key to preventing catastrophic complications such as ischemia, perforation, and sepsis.
